# Benefit from retrieval practice is linked to temporal and frontal activity in healthy young and older humans

**DOI:** 10.1093/texcom/tgac009

**Published:** 2022-02-17

**Authors:** Catherine-Noémie Alexandrina Guran, Lorena Deuker, Martin Göttlich, Nikolai Axmacher, Nico Bunzeck

**Affiliations:** Department of Psychology I, University of Lübeck, Maria-Goeppert-Straße 9a, Lübeck 23562, Germany; Vienna Cognitive Science Hub, University of Vienna, Kolingasse 14-16, Vienna 1010, Austria; Department of Neuropsychology, Faculty of Psychology, Institute of Cognitive Neuroscience, Ruhr University Bochum, Universitätsstraße 150, Bochum 44801, Germany; Department of Neurology, University Hospital Schleswig-Holstein, University of Lübeck, Ratzeburger Allee 160, Lübeck 23538, Germany; Center of Brain, Behavior and Metabolism (CBBM), University of Lübeck, Ratzeburger Allee 160, Lübeck 23562, Germany; Department of Neuropsychology, Faculty of Psychology, Institute of Cognitive Neuroscience, Ruhr University Bochum, Universitätsstraße 150, Bochum 44801, Germany; Department of Psychology I, University of Lübeck, Maria-Goeppert-Straße 9a, Lübeck 23562, Germany; Center of Brain, Behavior and Metabolism (CBBM), University of Lübeck, Ratzeburger Allee 160, Lübeck 23562, Germany

**Keywords:** aging, fMRI, long-term memory, retrieval practice

## Abstract

Retrieval practice improves retention of information in long-term memory more than restudy, but the underlying neural mechanisms of this “retrieval practice effect” (RPE) remain poorly understood. Therefore, we investigated the behavioral and neural differences between previously retrieved versus restudied items at final retrieval. Thirty younger (20–30 years old) and twenty-five older (50+ years old) adults learned familiar and new picture stimuli either through retrieval or restudy. At final recognition, hemodynamic activity was measured using functional magnetic resonance imaging (fMRI). Behaviorally, younger and older adults showed similar benefits of retrieval practice, with higher recollection, but unchanged familiarity rates. In a univariate analysis of the fMRI data, activation in medial prefrontal cortex and left temporal regions correlated with an individual’s amount of behavioral benefit from retrieval practice, irrespective of age. Compatible with this observation, in a multivariate representational similarity analysis (RSA), retrieval practice led to an increase in pattern similarity for retested items in a priori defined regions of interest, including the medial temporal lobe, as well as prefrontal and parietal cortex. Our findings demonstrate that retrieval practice leads to enhanced long-term memories in younger and older adults alike, and this effect may be driven by fast consolidation processes.

## Introduction

Compared to repeated study, retrieval practice, or testing of information, is a more beneficial approach to learning and retaining information (Abbott 1909; Spitzer 1939; Roediger III and Karpicke 2006; Karpicke and Roediger 2008; Karpicke and Blunt 2011). Despite a wealth of evidence from the psychological literature, the underlying neural processes, however, still remain unclear. In previous work, testing versus restudy increased activity in the striatum, precuneus and medial prefrontal cortex (mPFC, Herweg et al. 2018), hippocampus, lateral temporal cortices, and increased connectivity between hippocampus and mPFC (Wing et al. 2013). As such, the medial temporal lobe (Squire and Zola-Morgan 1991), but also the mPFC (Frey and Petrides 2000; Bunge et al. 2004; Xiang and Brown 2004) and parietal regions (see Wagner et al. 2005) have been identified as part of a core network underlying long-term memory encoding and retrieval (Rugg and Vilberg 2013). Importantly, these brain regions deteriorate with older age, especially temporal and frontal cortices (Hedden and Gabrieli 2004), and accordingly, declarative long-term memory is often impaired in older age (Nyberg et al. 2012). However, the retrieval practice effect (RPE) is not always reduced during healthy aging (Meyer and Logan 2013; Guran et al. 2019, 2020), which might be reflected by neural markers such as oscillatory alpha–beta power.

In terms of mechanistic explanations of the RPE, the fast route to consolidation (FRC) hypothesis (Antony et al. 2017) suggests that RP leads to a rapid online consolidation of information, circumventing longer processes including sleep consolidation. Further, retrieval would selectively enhance stimulus-specific neocortical networks and downregulate irrelevant connections, thereby leading to faster and more accurate future retrieval attempts. Importantly, retrieved information should quickly become hippocampus-independent and, therefore, rely more strongly on neocortical representations.

Direct empirical evidence in humans in favor of the FRC hypothesis is limited, with some studies finding increased activity in cortical areas (Eriksson et al. 2011), but only slow decreases in HC activity (Ferreira et al. 2019), or reduced activity for restudied vs retested stimuli (Keresztes et al. 2013). In a study not directly aimed at the RPE (Brodt et al. 2018), rapid, yet temporally stable, microstructural changes within the posterior parietal cortex in the context of a study/retrieval paradigm could be demonstrated (Brodt et al. 2016). These findings further suggest that plasticity-related changes in the neocortex, associated with the learning of new episodic information, are not necessarily limited to days, as many theories of systems consolidation suggest (McGaugh 2000; Frankland and Bontempi 2005); instead, retrieval may lead to fast trackable changes in neocortical activity or representations (Antony et al. 2017, see above). Our study aims to advance previous work regarding the RPE by looking at (i) neural underpinnings, such as activation or neocortical representations, of retrieval practice directly after learning and (ii) by investigating possible changes across the life span in behavior as well as at the neural level.

To address these questions, we used a previously established RP paradigm (Herweg et al. 2018; Guran et al. 2019, 2020), in a sample of younger (20–30 years of age) and older (51–77 years of age) healthy humans. Hemodynamic activity was measured using fMRI, while subjects performed a final remember/know recognition memory test on previously tested, or restudied, images randomly intermixed with unknown distractors. On a neural level, we employed fMRI with univariate analysis approaches in combination with state-of-the-art representational similarity analysis (RSA, Kriegeskorte et al. 2008; Dimsdale-Zucker and Ranganath 2018). While univariate fMRI analyses allow conclusions regarding increases or decreases in hemodynamic activity related to RP and RP benefits, RSA is a specific type of multivoxel pattern analysis (MVPA) and provides more direct insights into the representation of information, including memories, on the basis of correlations between pairs of distributed activity patterns (van Kesteren et al. 2016). Based on our own previous research, we expected the RPE to be driven by enhanced recollection in both age groups but reduced in the older participants. We also expected retested stimuli in a final retrieval test to be (i) less dependent on the hippocampus and temporal cortex, and (ii) to be more dependent on the neocortex. Both hypotheses were investigated in terms of activation, as well as representational similarity changes. Based on our previous findings of a neural marker in the oscillatory domain (Guran et al. 2019), we also expected a predictive neural marker for retrieval practice benefit, investigated via univariate regression analysis in the fMRI data, i.e., increased activity in brain areas related to memory (e.g. temporal lobe, frontal cortex), and in particular, consolidation related to increased RP benefits.

## Materials and methods

### Sample

Our sample comprised a total of 64 participants, 31 younger (20–30 years of age) and 33 older (51–77 years of age). They were recruited through the Online Recruitment System for Economic Experiments (Greiner 2015). Inclusion criteria were right-handedness, fluency in German, no personal history of neurological or psychiatric disorders, and MRI compatibility (no ferromagnetic implants). Additionally, we screened subjects in the older age group for cognitive impairment with the Montreal Cognitive Assessment Scale (MoCA, Nasreddine et al. 2005). Subjects with a MoCA score lower than 22 were excluded from the experiment (Freitas et al. 2013). We had to exclude nine participants from the initially measured sample (three subjects misunderstood the task, one had a MoCA score of <22 but was erroneously still invited to partake in scanning, one showed very poor performance, one had a brain abnormality discovered during scanning, one showed excessive head movements in the scanner). This reduced our sample to 30 younger (Mean age = 24.5 ± 3.1, 16 female) and 25 older (Mean age = 61.4 ± 6.7, 16 female) participants. All participants gave written informed consent prior to participation, and the study was approved by the ethics committee of the University of Lübeck, Germany.

### Experimental design

**Fig. 1:**
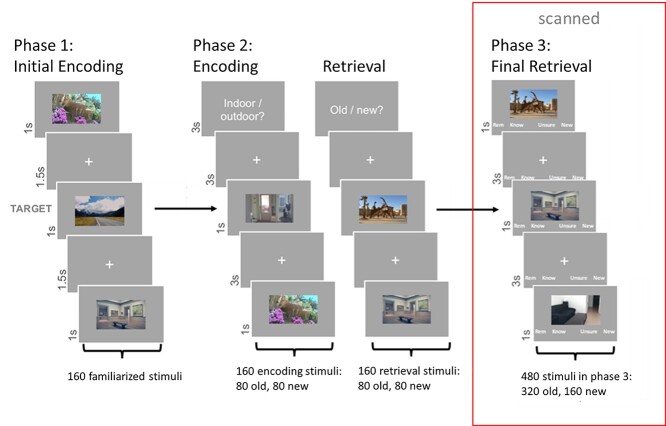
Experimental paradigm. In Phase 1, participants were familiarized with 160 RGB indoor and outdoor images by means of a target detection task. These stimuli, intermixed with 160 new stimuli, were presented in Phase 2 in a block design, randomly assigned to either a study task (indoor/outdoor categorization), or a retrieval practice task (old/new categorization). Finally, participants were brought into the scanner and shown all 320 items from Phase 2, intermixed with 160 new stimuli, in a final recall task. Participants had to categorize the stimuli as remembered, known, unsure, or new. Adapted with permission from Guran et al. (2020).

The experimental paradigm was almost identical to the paradigm described in Guran et al. 2020. Briefly, the experiment consisted of three phases (see Fig. 1). In Phase 1, participants were familiarized with 160 outdoor and indoor images (80 each) by means of a target detection task: the target stimuli (one indoor and one outdoor picture) were presented initially for 12 s. Subsequently, 160 images were presented three times each for 1 s in pseudorandom order intermixed with 9% of target trials (i.e., 48 target and 480 nontarget trials) to familiarize participants with them. Each image was followed by an interstimulus interval of 1.5 s (white fixation cross on gray background). Participants had 2 s to respond to the target stimuli with a button press and had the opportunity to pause every 96 trials. Target stimuli were not shown again outside of Phase 1.

In Phase 2, participants had to perform two different, randomly alternating tasks, while viewing 160 new stimuli randomly intermixed with the 160 familiarized stimuli. The tasks were designed to induce a restudy, and a retrieval context for half of the new and familiarized stimuli each. In the study task (STU), participants gave simple indoor/outdoor categorization judgments, using keyboard button presses. In the retrieval practice task (RET), participants gave an old/new recognition judgment, again through button presses. The combination of the two factors Task (STU/RET) and stimulus Novelty (OLD/NEW) resulted in a 2 × 2 repeated measures design with 80 stimuli per condition. Task blocks were 8 trials long, each block containing 4 OLD and 4 NEW stimuli in random order. Task (STU/RET) was also randomized across blocks. An instruction on the screen informed participants about the upcoming task prior to the start of each block. Images were presented for 1 s with an interstimulus interval of 3 s (fixation cross). Participants gave their response within 2.8 s using their right index and middle finger. Slower responses were coded as “no button press.” Response-button mappings were counterbalanced across participants. Participants could make a self-paced pause every 64 trials.

For Phase 3, participants were brought into the scanner and viewed the stimuli via a mirror reflecting a screen positioned at the back of the scanner. In the scanner, participants performed a surprise recognition task roughly 15–20 min after the end of the second phase (time to move from the behavioral lab to the MRI, fill in MRI safety questionnaires, and run the resting-state scan). The 320 previously encountered stimuli, counterbalanced for location (indoor/outdoor), stimulus Novelty, and Task in Phase 2, were intermixed with 160 unseen distractor images (i.e., 160 stimuli from the study task, 160 stimuli from the retrieval practice task, and 160 unseen distractors). Each image was presented for 1 s with four response options in German below the image: Remember—Know—Unsure—New (or in opposite order, depending on key mapping in Phase 2). Participants were instructed, orally and in writing, about the meaning of each response option. They were asked to choose “Remember” when they recognized a picture *and* could recollect specific thoughts or associations linked to the study episode (recollection). They were asked to choose “Know” when they recognized the picture but were not able to recall specific associations related to the study episode (familiarity). “Unsure” was to be pressed when they did not know whether a picture was old or new, and “New” when they were sure they had not seen the picture before. The jittered interstimulus interval was 3 s (fixation cross and response options, with a 1-s jitter, duration range 3–4 s) during which participants could still give their response using the scanner button boxes. Participants could make a self-paced pause every 60 trials. Phases 1 and 2 were performed consecutively, with small breaks (5–10 min) between them. Phase 3 was conducted in four functional runs, of circa 10 min each. Participants could do self-paced break between runs.

Images were randomly assigned to the different phases and conditions for each subject. To control for effects of illumination, mean luminance on each color channel (R,G,B) was set to 127 (scale from 0 to 288) and images were presented on a gray background of equal luminance. Prior to each phase of the experiment, participants completed a brief training session, introducing the participant to the upcoming task while using a training set of visual stimuli (not used for analysis). For Phase 3, participants practiced twice, once outside and once inside of the scanner. Images used during the training phase were not shown again.

### Statistical analyses of behavioral data

Behavioral responses were analyzed as in our previous work (Guran et al. 2019, 2020). To reiterate, accuracy of behavioral responses was assessed on the basis of signal detection theory (Stanislaw and Todorov 1999). For the target detection task during Phase 1, hits were defined as correctly detected targets. For the old/new categorization task during Phase 2, hits were defined as old stimuli correctly classified as old. For the indoor/outdoor categorization task during Phase 2, hits were defined as correctly classified indoor images. For Phase 3, hits were defined separately for old stimuli that were remembered, and old stimuli that were recognized, but not remembered (Known). False alarm rates (FA) were also calculated separately for each response option: Know-FA was calculated as the proportion of new items that were erroneously identified as familiar, while remember-FA was the proportion of new items that were erroneously remembered. Specifically, *d*′ (*d*-prime) was calculated by subtracting the inverse phi (conversion of probabilities into *z*-scores according to the normal cumulative distribution function) of the hit rate from the inverse phi of the false alarm rate for each subject and condition. As the inverse phi of 0 and 1 is -∞ and ∞ respectively, 0.5 was added to the number of hits and false alarms and 1 was added to the number of signal and no signal trials (Stanislaw and Todorov 1999).

In Phase 3, behavioral data were analyzed using a 2 (Memory: Remember vs Know) × 2 (Task: Study vs Retrieval) × 2 (stimulus Novelty: old vs new) × 2 (Age: young vs older, between subjects) repeated measures (Bayesian) ANOVA, and post-hoc tests were conducted on interactions using *T*-tests, or Mann–Whitney U tests, where appropriate and necessary. Alpha-levels were Bonferroni corrected for multiple comparisons, when appropriate (corrected alpha-thresholds reported). Calculation of *d*′ and hit rates was performed in MATLAB 2019a (The MathWorks; RRID:SCR_001622), while the Bayesian analysis of behavioral data was performed with JASP (JASP Team 2019). Barplots were made using MATLAB and the Gramm toolbox (Morel 2018).

### Image acquisition and preprocessing

Structural and functional MR imaging was performed at the University of Lübeck, CBBM Core Facility Magnetic Resonance Imaging, using a 3-T Siemens Magnetom Skyra scanner equipped with a 64-channel head coil. Functional images were acquired applying a single-shot gradient-recalled echo-planar imaging (GRE-EPI) sequence sensitive to blood oxygen level dependent (BOLD) contrast (TR = 1650 ms; TE = 25 ms; flip angle = 75°; voxel resolution 2.5 × 2.5 × 2.5 mm^3^; 80 × 80 matrix; 60 transversal slices; GRAPPA factor 2 and simultaneous multislice factor 2). We recorded 4 runs with 345 volumes each (plus 6 dummy scans). Structural images of the whole brain using a 3D T1-weighted MP-RAGE sequence were acquired (TR = 1900 ms; TE = 2.44 ms; TI = 900 ms; flip angle 9°; 1 × 1 × 1 mm3 resolution; 192 × 256 × 256 mm^3^ field of view; acquisition time 4.5 min). Additionally, we acquired resting-state runs of each participant (TR = 1650 ms; TE = 25 ms; flip angle = 80°; voxel resolution 2.5 × 2.5 × 2.5 mm^3^; 80 × 80 matrix; 60 transversal slices; GRAPPA factor 2 and simultaneous multislice factor 2); however, those data will not be presented in this article. Data were preprocessed using SPM 12 (http://www.fil.ion.ucl.ac.uk/spm/software/spm12/, RRID: SCR_007037SCR_007037) and MATLAB. EPIs were slice time corrected and realigned to correct for head motion. T1 images were coregistered onto mean EPIs. All images were normalized to MNI space based on normalization parameters derived from a segmentation of T1-weighted images into white matter, gray matter, and CSF using default tissue probability maps. Smoothing was performed with a 6 mm full width at half maximum (FWHM) Gaussian kernel. As mentioned above, one participant was excluded due to excessive head motion (exceeding 4 mm in each run). For the RSA, preprocessing followed a similar pipeline but neither normalization nor smoothing was performed on the functional data. In addition, T1 images (mapped onto mean EPIs) were segmented using FreeSurfer (Fischl 2012).

#### Analysis of functional MRI data

MRI data were analyzed using SPM12 (http://www.fil.ion.ucl.ac.uk/spm/software/spm12/, RRID:SCR_007037) and custom MATLAB scripts, in case of the RSA. For the univariate analysis in SPM, first- and second-level analyses were performed. On the first level, we included regressors for the levels of *Task* (study vs retrieval) and *Novelty* (old vs new, i.e., images that were repeatedly presented or only once during the retrieval practice task in Phase 2), resulting in the conditions study-old, study-new, retrieval-old, and retrieval-new. Correct rejections, false alarms, unsure responses, and errors were also modeled on the first level, but not included in the analysis on the second level. The factor memory type (remember vs know responses) could not be included due to insufficient trial numbers in each condition (see Supplementary Tables S1 and S2), which would have led to a post-hoc exclusion of a substantial amount of participants (minimum 10, with very liberal thresholds, 30 with a more conservative approach of excluding each participant that had below 10 trials in one of the conditions). Therefore, we averaged the hemodynamic responses across remember and know trials (both for hits and false alarms). We used the “FAST” setting for temporal autocorrelation modeling (prewhitening, Bollmann et al. 2018; Olszowy et al. 2019). Data were high-pass filtered (128 s) and motion parameters were included in the model. The four functional runs were concatenated using the inbuilt spm_concatenate function, and the data were fitted to the canonical hemodynamic response function (HRF).

On the second level, contrasts of interest were entered into a flexible factorial design with the within-subjects factors task (study vs retrieval) and novelty (old vs new), and the between-subjects factor age group (young vs older) (see Gläscher and Gitelman 2008). The uncorrected cluster-forming threshold for all analyses was *P* < 0.001, with a minimum of 50 voxels. Clusters of at least 50 voxels, below a familywise error-corrected *P*-value of 0.05 were considered significant (whole brain, disregarding activations in the cerebellum as we had no particular hypothesis for this part of the brain). We selected this cluster extent based on our primary focus of cortical areas, not warranting smaller cluster sizes, which in turn can lead to reporting of spurious effects (Woo et al. 2014).

For the multivariate RSA, we calculated weighted 3d images for each trial, based on stimulus onset (weighted mean between volumes, with a temporal lag of 5 s to account for the hemodynamic response peak). Systematic effects of motion were regressed out (using the glmfit function in MATLAB). The preidentified regions of interest (hippocampus, entorhinal cortex, parahippocampal cortex, temporal cortex, frontal cortex, and parietal cortex; see below *RSA—multivariate mixed model*) were defined with FreeSurfer segmented maps, and data from each ROI were extracted from each trial’s image. Furthermore, we conducted an analysis subdividing the frontal cortex into dorsolateral prefrontal cortex (dlPFC), inferior frontal gyrus (IFG), orbitofrontal cortex (OFC), and caudal anterior cingulate (caudal ACC), to further investigate the role of the frontal cortex in retrieval practice. As we were interested in the effects of retrieval practice on cortical representations and, more specifically, whether similarity between stimuli changed, we correlated voxel patterns of every trial with voxel patterns of every other trial across all voxels of a given ROI, for every participant separately. We only included between-run correlations. Then, the correlations from different conditions were averaged across trials. Data were subsequently analyzed across participants in a linear mixed multilevel model in RStudio, Version 1.1.463 (R Core Team, 2018), using the package lmer, which allows to account for additional covariates, such as participants. Following a stepwise inclusion procedure, we first added random effects (RE) and compared the model to the null model (no predictors) and then continued adding fixed effects (FE), comparing each new model to the previously best one, using likelihood-ratio tests. FEs were added in order of their conceptual importance regarding our hypotheses, interactions being added after adding relevant main effects.

### Data availability

All data are available upon reasonable request via email, including a formal project outline, from the corresponding author.

## Results

### Behavioral results

#### Phase 1

In Phase 1, we compared accuracy (*d*′) and reaction times (RTs) in the target detection task between young and older participants in a two-sample *T*-test (Homogeneity of Variance was present in both cases, *P* > 0.1). On average, accuracy was high in both age groups, and there was no significant difference in performance between young and older adults (*P* > 0.4), *d′*_young_ = 5.43 ± 0.3; *d′*_older_ = 5.36 ± 0.38 (mean *d′* ± SD). Similarly, there was no significant difference in RT between both groups, only a trend for older participants to be slower, *t*_(53)_ = −1.96, *P* = 0.055, RT_young_ = 556 ± 71 ms, RT_older_ = 594 ± 71 ms (mean RT ± SD).

#### Phase 2

For Phase 2, we analyzed *d′* in a 2 × 2 repeated measures ANOVA with task (study vs retrieval) as within-subjects factor and age (young vs old participants) as between-subjects factor. Note that the factor novelty cannot be analyzed here (based on how hits etc. were defined through signal detection in Phase 2). For RTs, we conducted a 2 × 2 × 2 ANOVA including the within-subjects factors task and novelty (old vs new stimuli), and the between-subjects factor age. We tested assumptions of normality of residuals with Kolmogorov–Smirnov tests and homogeneity of variance with the Levene test. As some assumptions (homogeneity of variance, normality of residuals) were violated for some variables, we conducted Bayesian repeated measures ANOVAs alongside the traditional frequentist analysis. In terms of memory accuracy (see Table 1), the frequentist 2 × 2 ANOVA revealed a main effect of task (*F*_(1, 53)_ = 270.89, *P* < 0.001, η^2^ = 0.84), but no main effect of age and no interaction of age × task (*P* > 0.5). A post-hoc *T*-test revealed that accuracy was lower in the retrieval task as compared to the study task (*t*_(54)_ = 16.55, *P* < 0.001). The Bayesian ANOVA confirms this result, suggesting that the best model includes the factor task (BF_10_ = 4.84e+28).

**Table 1:** Response accuracy (*d*′) in Phase 2.

Age group	Task	Mean	SD	*N*
Young	Study	3.803	0.702	30
	Retrieval	1.795	0.829	
Older	Study	3.889	0.527	25
	Retrieval	1.712	0.701	

For reaction times (see Table 2), there were main effects of task (*F*_(1, 53)_ = 144.68, *P* < 0.001, η^2^ = 0.73) and novelty (*F*_(1, 53)_ = 16.86, *P* < 0.001, η^2^ = 0.24), and an interaction of task x novelty (*F*_(1, 53)_ = 5.81, *P* = 0.019, η^2^ = 0.1) in the frequentist ANOVA. All other effects were not significant (*P* > 0.1). Post-hoc *T*-tests revealed that participants were faster in the study (compared with retrieval) task (*T*_(54)_ = −12.07, *P* < 0.001), and faster for old (compared with novel) stimuli (*T*_(54)_ = −4.17, *P* < 0.001). In the Bayesian ANOVA, the best model included both factors task and novelty (BF_10_ = 2.74e+37), but not the interaction of task x novelty (BF_inclusion_ task x nov = 0.58). Similarly as in the frequentist results, descriptively, participants were slower in the retrieval task, as well as for new items.

**Table 2:** Reaction times (in seconds) in Phase 2.

Age group	Task	Novelty	Mean	SD	*N*
Young	Study	Old	0.762	0.14	30
		New	0.769	0.15	
	Retrieval	Old	0.886	0.19	
		New	0.917	0.195	
Older	Study	Old	0.819	0.119	25
		New	0.825	0.121	
	Retrieval	Old	0.959	0.147	
		New	0.988	0.134	

#### Phase 3

In Phase 3, we analyzed *d*′ in a 2 × 2 × 2 × 2 ANOVA, with memory (remember vs know), task (study vs retrieval), novelty (old vs new in Phase 2), and age (young vs older participants) as factors. Homogeneity of variance (Levene test) was present in all but one variable (retrieved new items that were rated as “known”), and normality of residuals (Kolmogorov–Smirnov tests) was present in all but three variables (retrieved old items that were rated as known in older participants, encoded old items that were rated as known by older participants, and retrieved old items that were rated as remembered by young subjects). Because of the large sample size of *n* = 55 subjects in total, we still conducted frequentist ANOVAs, but we also conducted a Bayesian repeated measures ANOVA. In the Bayesian analysis, the best model (BF_10_ = 3.3e+100) mirrored the main effects found in the frequentist ANOVA described below, as well as the interaction of memory by novelty. A model that also included the interaction of memory by task also performed very well (BF_10_ = 2.55e+100).

##### Main effects

**Fig. 2:**
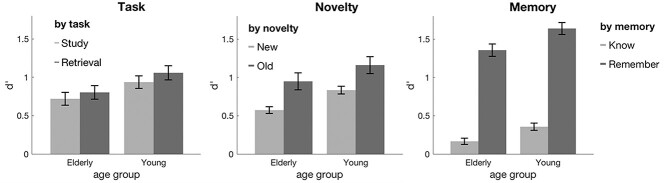
Main effects of task, novelty, memory, and age group on memory accuracy in Phase 3. Retrieval practice led to higher memory accuracy (task), and old stimuli were recognized more accurately (novelty). Overall, stimuli elicited more recollection than familiarity (memory), and younger participants outperformed older ones (across all graphs, but not significant in a post-hoc *T*-test). Error bars reflect standard error of the mean (SEM).

The frequentist ANOVA revealed main effects of memory (*F*_(1, 53)_ = 161.83, *P* < 0.001, η^2^ = 0.75), task (*F*_(1, 53)_ = 34.37, *P* < 0.001, η^2^ = 0.39), novelty (*F*_(1, 53)_ = 131.4, *P* < 0.001, η^2^ = 0.71), and age (*F*_(1, 53)_ = 6.59, *P* = 0.013, η^2^ = 0.11). Furthermore, post-hoc *T*-tests revealed higher recollection than familiarity rates (*T*_(54)_ = 12.9, *P* < 0.001), higher memory scores for old items (*T*_(54)_ = 12.05, *P* < 0.001), and, importantly, higher memory scores for items previously encountered in the retrieval task (retrieved items; *T*_(54)_ = −7.25, *P* < 0.001, see Fig. 2). Older participants showed a trend for lower memory scores than younger ones (*T*_(53)_ = 1.49, *P* = 0.071); thus, despite the significant main effect of age in the ANOVA, the effect in the post-hoc *T*-test was only at trend level (one-sided two-sample *T*-test; see Fig. 2).

##### Interactions

**Fig. 3:**
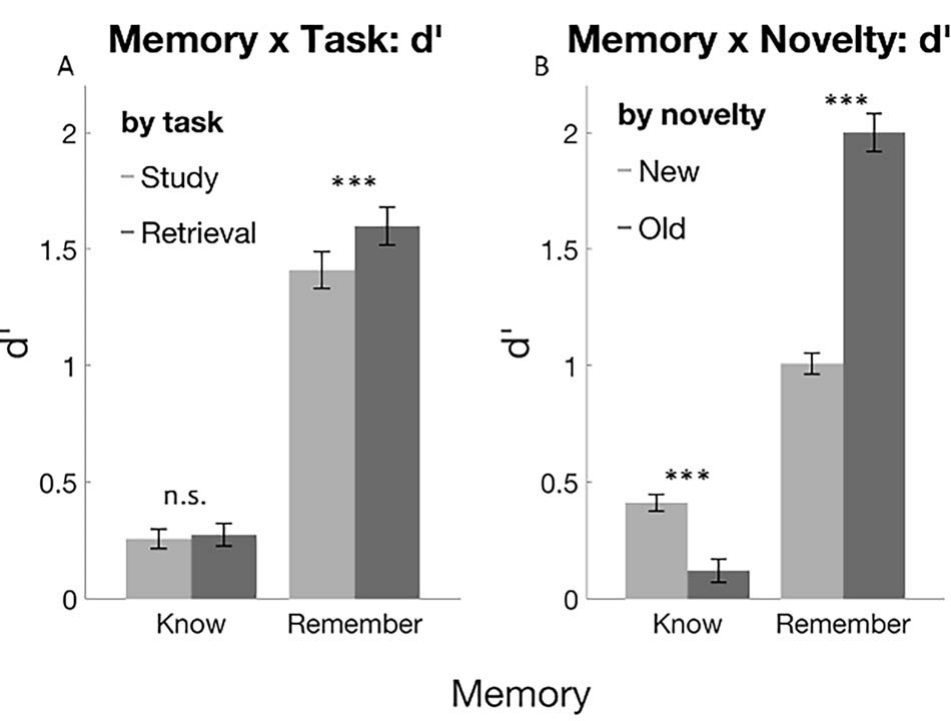
A) Interaction of memory type by task. Retrieval practice significantly increased remember responses, i.e., recollection rates, but it had no effect on familiarity. ^*^^*^^*^P < 0.001. B) Interaction of memory by novelty. Remember responses were associated significantly more with old items than new items, while know responses were associated significantly more with new items than old items. ^*^^*^^*^P < 0.001. Error bars show SEM.

There was a significant interaction of memory by task (*F*_(1, 53)_ = 17.58, *P* < 0.001, η^2^ = 0.25), which was driven by a significant difference between study and retrieval for remembered items, but not for known items (see Fig. 3A; *T*_(54)_ = 7.0, *P* < 0.001, and *T*_(54)_ = 0.72, *P* > 0.4, respectively). There was a disordinal interaction between memory and novelty (*F*_(1, 53)_ = 106.23, *P* < 0.001, η^2^ = 0.67); old items were associated with more recollection and fewer familiarity responses (see Fig. 3B; *T*_(53)_ = −10.0, *P* < 0.001, and *T*_(48)_ = −4.27, *P* < 0.001, respectively). There were no other significant interactions, including task by age (all *P* > 0.05).

It should be noted that behavior in Phase 3 was analyzed independent from responses in Phase 2 since previous studies of the retrieval practice effect show that even unsuccessful retrieval attempts lead to improved memory (see Jacoby et al. 2005; Guran et al. 2019, 2020). This also ensured the highest possible number of trials per condition. In addition to *d*′, we also analyzed our data on the basis of corrected hitrates. Similar to our previous studies (see Guran et al. 2019; Guran et al. 2020), it revealed comparable results (in terms of direction and significance) to the *d*′ results.

### fMRI results

#### Flexible factorial design—univariate analysis

The 2 × 2 × 2 ANOVA with the within-subjects factors task (study vs retrieval), and novelty (old vs new in Phase 2), and the between-subjects factor age (young vs old) revealed main effects of age and novelty. The age effect (contrast older > younger) was associated with widely distributed higher activity for older adults in the bilateral precentral/postcentral gyrus, medial and lateral PFC, temporal cortex, basal ganglia, bilateral thalamus, temporal cortex, and supramarginal gyrus (see Supplementary Fig. S1 and Supplementary Table S3). For the opposite contrast (young > older), there was higher BOLD activity in the right frontal inferior operculum, left temporal pole and middle temporal gyrus, HC, parahippocampal cortex, fusiform gyrus, and lingual gyrus (see Supplementary Fig. S2 and Supplementary Table S4). The main effect of novelty was driven by stronger activation in central superior motor areas (left and right) for new stimuli in comparison to old ones (see Supplementary Fig. S3 and Supplementary Table S5). There were no significant clusters for the factor task (pFWE = 0.082, RET > STU, right supramarginal gyrus), or any of the possible interactions. For a discussion of these results, see Supplementary Materials.

#### Link between behavior and brain activation—regression analysis

**Fig. 4:**
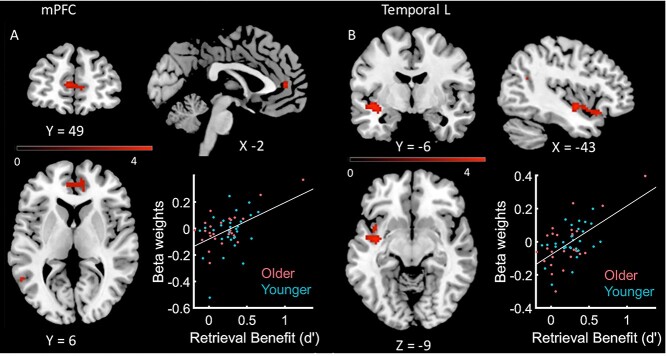
Multiple regression between retrieval benefit (*x*-axis) and activity in mPFC/cingulate A) and left temporal cortex B), respectively. RP increased activity in both areas; see also Table 3. All activation maps are thresholded at *P* < 0.05 (FWE-corrected at cluster-level using a cluster-forming threshold at voxel level of *P* < 0.001). Beta weights extracted from cluster.

We performed a multiple regression analysis, with retrieval benefit (*d*′_Retrieval_—*d*′_Study_ in Phase 3) as a predictor for the difference in brain activation of retrieval versus study (calculated on the first level). In this analysis, we pooled young and older participants (i) since there was no significant interaction between age and task at the behavioral level; (ii) while the behavioral main effect of task was very strong, the main effect of age only reached trend levels in the post-hoc *T*-test; and (iii) since the focus of this work was on the retrieval practice effect, while age was of secondary importance. We found three significant clusters (see Table 3), one in the superior temporal pole, one in the mPFC (including the medial frontal gyrus), and one in the middle occipital gyrus, see Fig. 4. One outlier with particularly high RP benefit was identified visually and statistically (see Fig. 4, RP benefit value >Mean + 3 SD). Correlations in both clusters stayed significant in a Pearson correlation after excluding this subject (both *P* < 0.025).

**Table 3:** Areas with higher activity in dependence of an individual’s benefit from retrieval practice.

				Statistics			
Cluster location	Peak coordinates (MNI)			Cluster		Peak	
	*x*	*y*	*z*	*P* (FWE-corr)	# of voxels	*P* (FWE-corr)	*T*
mPFC/anterior cingulum R + L	6	53	12	0.042	73	0.381	4.63
Superior temporal pole L, insula L	−44	−2	−8	0.003	129	0.395	4.61
Middle occipital gyrus R	28	−82	15	0.039	74	0.853	4.12

The larger the RP benefit, the stronger the activation in each of the areas.

#### RSA—multivariate mixed model

As described in the introduction, we hypothesized that through retrieval of information, memory traces should become less dependent on the HC and more dependent on the neocortex. We performed our multivariate RSA in two ways: first, focusing on the differences between the hippocampal formation and cortical areas (temporal, frontal, and parietal cortex). The parietal cortex ROI included the supramarginal gyrus and precuneus (Lee et al. 2019, lateral and medial: Jonker et al. 2018, Precuneus: Brodt et al. 2018) the frontal cortex ROI included the entire frontal gray matter except for the precentral gyrus; the temporal cortex ROI included the temporal pole and inferior temporal gyrus; see Supplementary Fig. S4.

Second, we investigated differences between the hippocampal formation and 12 other ROIs (some contained within the cortical areas of the other analysis); see in Table 4. All models were estimated on unambiguous/even data from correlations within-level of each factor, e.g. study-old with study-old stimuli, from between different runs.

**Table 4:** All subdivided ROIs (left column) and their contributions to the ROI areas (right column).

ROI	Part of ROI area
Hippocampus	Hippocampus
Precuneus	Parietal cortex
Supramarginal gyrus	Parietal cortex
Temporal pole	Temporal cortex
Inferior temporal gyrus	Temporal cortex
Lateral orbitofrontal cortex	Frontal cortex
Medial orbitofrontal cortex	Frontal cortex
Middle frontal gyrus	Frontal cortex
Superior frontal gyrus	Frontal cortex
Entorhinal cortex	/
Parahippocampal gyrus	/
LOC	/
Temporal gyrus	/

/, ROIs were not included in any ROI area.

##### Random and fixed effects

The mixed-model analyses revealed participant as a significant random effect (*X*^2^ = 70.5, *P* < 0.0001, dfs = 1) for pattern similarity (i.e., correlation values extracted for each ROI). In terms of fixed effects, the model including task had a significantly better fit (*X*^2^ = 4.56, *P* = 0.033, dfs = 1) for explaining the pattern similarity data. ROI area (frontal, parietal, temporal, HC) also improved the model significantly (*X*^2^ = 39.72, *P* < 0.0001, dfs = 3). Neither stimulus novelty (*P* > 0.5) nor age (*P* > 0.4) as fixed effects improved the model significantly in comparison to the more parsimonious random effects model. The model including both Task and ROI as fixed effects (AIC = −795,704) slightly outperformed either model containing only one of the factors (*X*_Task_^2^ = 54.13, *P* < 0.0001, *X*_ROI_^2^ = 14.74, *P* = 0.001). There were no significant 2-, 3-, or 4-way interactions (all *P* > 0.15).

##### Random slopes and Post-Hoc Tests

We tested for random slopes of Task and ROI. Including Task as a random slope significantly improved model fit (*X*^2^ = 14.76, *P* < 0.001, dfs = 2, AIC = −795,715): the final model thus included participant as random effect, ROI area and Task as fixed effects, and Task as random slope.

**Fig. 5:**
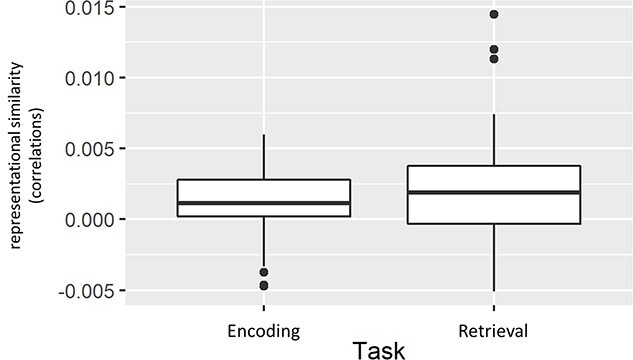
Boxplot for representational similarity in the restudy and retrieval tasks. Data were averaged across regions and participants. Dots mark outliers beyond 1.5 SD.

To calculate post-hoc *T*-tests to investigate the direction of the fixed effects, we had to average across participants, thus decreasing power. Our two-sided *T*-test for the fixed effect of Task did not show a significant difference, *P* = 0.15. However, visual inspection of the fixed effect of Task (see Fig. 5) shows that, on a descriptive level, stimuli in the retrieval task elicited stronger representational similarity (the effect persisted when removing outliers outside of 3 SD).

For the fixed effect of ROI area, *T*-tests revealed significant differences between the HC and all other cortex areas as well as differences between the temporal, and the other cortices (frontal and parietal; see Table 5).

**Table 5:** Results from paired *T*-tests.

	Parietal		Frontal		Temporal	
	*T*	*P*	*T*	*P*	*T*	*P*
HC	−6.92	<0.0001^*^^*^^*^	−5.41	<0.0001^*^^*^^*^	−4.03	<0.0001^*^^*^^*^
Parietal	—	—	1.25	0.21 n.s.	3.72	0.0002^*^^*^
Frontal	—	—	—	—	1.99	0.046^a^

All dfs = 107,429. Bonferroni-corrected alpha for multiple comparisons: 0.00833.

n.s. not siginificant. ^a^Not significant after Bonferroni-correction. ^*^^*^P < 0.001. ^*^^*^^*^P < 0.0001.

**Fig. 6:**
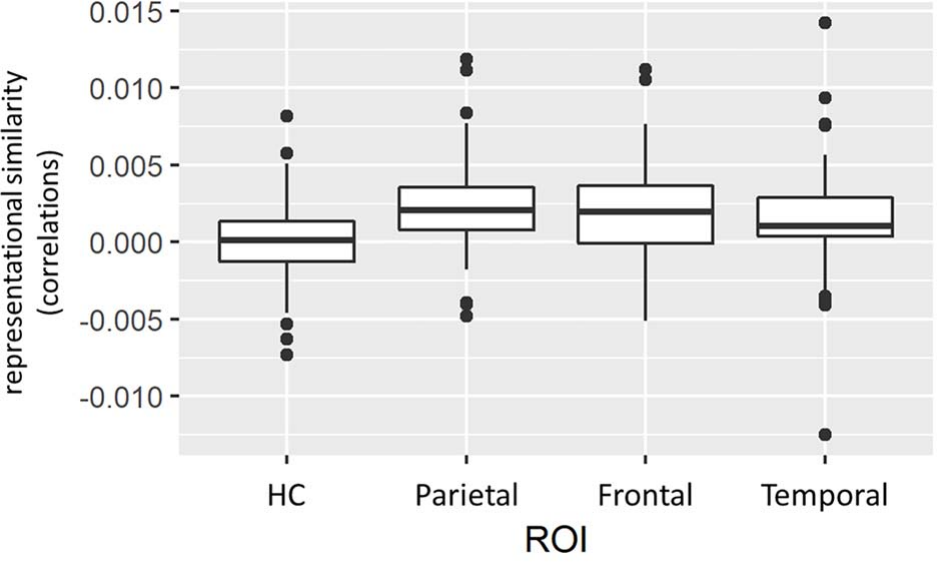
Boxplot for representational similarity in the different ROI areas. Data were averaged across participants and task conditions. Dots mark outliers beyond 1.5 SD. HC = hippocampus.

Cortical regions had higher representational similarity than the hippocampus, and the parietal cortex and frontal cortex also had higher representational similarity than the temporal cortex (see Fig. 6). However, the difference between frontal and temporal cortex fails to be significant after controlling for multiple comparisons.

Together, across ROIs, retrieval (vs restudy) was associated with higher mean similarity with no significant differences between both age groups (Fig. 5) suggesting that retrieval leads to a more similar neural representation of information. When comparing ROIs, on the other hand, the hippocampus shows significantly lower mean similarity independent of task condition (i.e., restudy/retrieval), suggesting that cortical regions exhibit a more similar neural representation of information in both tasks.

##### Results for subdivided ROIs

The results for the subdivided ROIs mirrored the results for the ROI areas. The final model includes participant as a random effect, Task and ROI as fixed effects, and a random slope of Task (*X*^2^ = 74, *P* < 0.0001, dfs = 2). No interactions contributed to the model fit.

##### Task *×* ROI post-hoc tests

To further investigate a possible interaction of Task and ROI area in line with the FRC hypothesis, we performed post-hoc *T*-tests, which yielded nonsignificant results (*P* > 0.9).

##### Exploratory correlation analysis

In analogy to the regression analysis in the univariate approach, we investigated a possible correlation between individual behavioral retrieval practice benefit and individual random slopes of task. There was no significant correlation (*P* > 0.4).

## Discussion

While the RPE has been studied for over a century, our understanding of its underlying neural mechanisms is incomplete. Using fMRI, we investigated how previously retested and restudied items differ at final recall. Retrieval practice increased recollection-based but not familiarity-based recognition memory, in both young and older adults. In a univariate analysis of the fMRI data, the degree of RP benefit was correlated with activity in the mPFC/anterior cingulate, temporal pole, and superior temporal gyrus, irrespective of age group. In line with this observation, a multivariate RSA revealed enhanced pattern similarity following retrieval within memory-related brain regions, including the medial temporal lobe, as well as frontal and parietal cortex. Additionally, pattern similarity was more pronounced within the neocortex (i.e., frontal and parietal lobe) as compared to the medial temporal lobe, including the hippocampus. As such, our findings provide novel insights into the underlying mechanisms of RP across the life span and they are compatible with the notion that the beneficial effects of retrieval practice on recognition memory can—at least in part—be explained by fast changes in neocortical representations.

As hypothesized, retrieval of information increased recollection-based recognition memory in both younger and older adults (Fig 3a). Importantly, this effect could be observed on a very short time scale of about 20 min after retrieval (versus study), which is compatible with our predictions derived from the FRC hypothesis (Antony et al. 2017). It suggests that RP leads to a rapid consolidation of memory (i.e., strengthening of memory content) without the need for slower processes, including sleep-related consolidation (Antony et al. 2017; see below for further explanation). The specific effect of RP on recollection-based memory follows research on the RPE (Verkoeijen et al. 2011; Guran et al. 2020) and provides empirical evidence not only for the FRC hypothesis (Antony et al. 2017) but also for the “Episodic Context Account” (ECA, Karpicke et al. 2014). According to the ECA, each retrieval attempt is associated with a reinstatement and update of episodic contexts, leading to enhanced recollection-based recognition memory, in line with our finding of increased recollection rates. According to dual-process models (Yonelinas et al. 1996; Yonelinas et al. 2010), recognition can be associated with specific details or associations of the study episode and then lead to recollection or induce familiarity in the absence of such recollective experience. Further support for dual-process models comes from functional imaging studies, suggesting that the hippocampus and posterior parahippocampal gyrus are closely associated with recollection, whereas the anterior parahippocampal gyrus is more associated with familiarity judgments (Diana et al. 2007). Our fMRI data do not allow us to further address this point, since we had to average across remember and know responses due to insufficient numbers of trials per condition (see below).

Interestingly, the RPE was present irrespective of stimulus novelty in Phase 2. That means, not only familiar but also novel stimuli that were shown in the context of retrieval were remembered better as compared to novel items shown in the restudy context. This is compatible with previous work (Jacoby et al. 2005) including our own (Herweg et al. 2018; Guran et al. 2019, 2020), suggesting that not successful retrieval but retrieval mode per se boosts memory performance (Rowland 2014). Whether this effect relates to enhanced effort (Pyc and Rawson 2009; Rowland 2014) or other processes such as fast mapping (Sharon et al. 2011) remains to be investigated.

At the neural level, a multiple regression analysis across all participants showed that the RPE was correlated with activity in the mPFC, anterior cingulate cortex, left superior temporal gyrus, and temporal pole, with stronger activation in these areas relating to larger RP benefits on an individual level. According to traditional models of systems consolidation, the hippocampus initially stores novel information; over time, through postlearning reactivation in resting periods and sleep, this information is being transferred to and stored within the neocortex including the PFC (McGaugh 2000; Frankland and Bontempi 2005; Deuker et al. 2013; Zhang et al. 2018). Importantly, this view has recently been challenged by the observation of rapid and temporally stable microstructural changes within the human posterior parietal cortex after learning (Brodt et al. 2018). Moreover, the mPFC is not only involved in sleep-related consolidation processes (Euston et al. 2007) but also in the retrieval of recent and remote memories (Gonzalez et al. 2013). Therefore, neocortical brain regions, including the parietal cortex and mPFC, appear to be an essential part of a rapid learning system. Our data further extend and specify this view by demonstrating a close relationship between neocortical brain regions (including the mPFC) and the positive effects of retrieval practice. The superior temporal gyrus, on the other hand, is essential for visual recognition memory (Nakamura and Kubota 1995), as tested here, and its anterior parts for semantic processing (Tsapkini et al. 2011; Visser and Lambon Ralph 2011). While this latter observation may fit to the Elaborative Retrieval Hypothesis (Carpenter 2009, 2011), according to which RP enhances semantic elaboration and improves memory accuracy, it is, at first glance, at odds with the fact that our RPE was driven by enhanced episodic-like (remember rates) but not semantic-like memory (familiarity rates). However, episodic and semantic information processing are not independent, and semantic elaboration, therefore, may positively influence episodic memory (Staresina et al. 2009).

While our goal was to analyze recollection and familiarity separately, based on remember and know responses, to further investigate contributions from episodic memory, this was not possible due to a large proportion of participants not providing enough responses in at least one of the categories. As individuals show large biases in responding with either “remember” or “know” (see also Dougal and Rotello 2007; Rotello et al. 2005 for the role of bias in recollection), studies aiming to investigate these differences in particular should include much larger samples in the future or remove the option of “unsure” responses. This, however, should be critically evaluated, since the removal of the unsure response option might unintentionally increase know responses. In addition, since we do not know what cognitive evaluations contributed to the unsure responses, it is not possible to retroactively average across unsure and know responses.

The multivariate RSA provides some preliminary evidence that RP leads to rapid changes in the neural representation of information. Specifically, items previously encountered in a retrieval context were, after circa 20 min, significantly more similar in their neural representations in a priori defined regions of interest (Fig. 5), as compared with items that were encountered in the study task. Importantly, this effect was (statistically speaking) independent of ROI and, therefore, observed across all cortical and subcortical regions, including the frontal, parietal, and temporal lobes, as well as the hippocampus. This was unexpected, since we hypothesized a dissociation through retrieval practice in prefrontal and parietal regions as compared to the MTL, as suggested by the FRC hypothesis. Therefore, our findings are only partly compatible with the FRC hypothesis, and they suggest that immediate retrieval of retested information is not completely hippocampus-independent but rather relies on changes in neural representations across the brain. To further investigate this issue, future studies should adopt a similar procedure as used here while simultaneously and systematically varying the length of the retention interval between retrieval (Phase 2) and final recall (Phase 3). In any case, rapid increases in pattern similarity in frontal, parietal, and, to an extent, temporal brain regions might reflect the integration of information into existing cortical networks and therefore underlie the retrieval practice effect. This is further supported by overall higher pattern similarity (i.e., irrespective of task) within frontal and parietal regions as compared to the HC (Fig. 6), since preexisting memory networks (or schemas) should be primarily represented in neocortical areas (Tse et al. 2007; Van Kesteren et al. 2012).

Contrary to our hypothesis and own previous work (Guran et al. 2019, 2020), the RPE did not differ between age groups (no interaction of task by age, Fig. 3A). In other words, although older adults had overall reduced memory scores in comparison to their younger counterparts, their RPE was unimpaired. One explanation is that this fMRI study had specific inclusion and exclusion criteria (see Materials and methods Sample), which may have led to a specific sample of healthy and high-functioning older adults. This interpretation is also supported by the absence of robust age-dependent differences in the simple target detection task of Phase 1. At the neural level, the absence of age-related impairments in the RPE was paralleled by equally enhanced representational similarity through retrieval in both age groups (i.e., there was no significant interaction between task and age). Similarly, there were no age effects in the RS analysis.

Finally, direct changes in representational similarity from one phase to the next cannot be assessed in an experimental design that does not measure neural activity at each phase of the paradigm. However, as stimuli were randomized for novelty and task across participants, and the only difference between the stimuli in the contrast “restudy vs retrieval practice” was whether they had been retrieved or restudied in Phase 2, it is extremely unlikely that the observed differences between restudy and retrieval stimuli arose from a different source than the task condition itself.

In summary, the retrieval practice effect is characterized by rapid and specific increases in recollection-based recognition memory and changes in neural activation in both young and older adults. In particular, activity in the mPFC/anterior cingulate and left anterior temporal lobe predicted the increase in recognition memory through retrieval, suggesting an involvement of brain regions previously associated with semantic processing, fast learning, and memory consolidation. Furthermore, RP increased pattern similarity, and pattern similarity was generally higher within the frontal cortex, parietal cortex, and temporal cortex. As such, our data extend current theoretical views of the RPE and provide empirical evidence that retrieval practice involves fast changes in neocortical representations of information.

## Supplementary Material

Guran_etal_CCC_SuppMat_Feb22_tgac009
