# Supplementary material for: Benefit from retrieval practice is linked to temporal and frontal activity in healthy young and older humans
Source: Cereb Cortex Commun. 2022 Feb 17;3(1):tgac009. doi: 10.1093/texcom/tgac009 (PMC8966694; doi:10.1093/texcom/tgac009)
Supplement: Guran_etal_CCC_SuppMat_Feb22_tgac009 [file guran_etal_ccc_suppmat_feb22_tgac009.docx]

Supplementary Materials

**Benefit from retrieval practice is linked to temporal and frontal activity in healthy young and older humans**

C.-N. Alexandrina Guran^1,2^, Lorena Deuker^3^, Martin Göttlich^4^,

Nikolai Axmacher^3^, Nico Bunzeck^1,5^

**Results – GLM**

The age effect (contrast older > younger) was associated with widely distributed higher activity for older adults in the bilateral precentral/postcentral gyrus, medial and lateral PFC, temporal cortex, basal ganglia, bilateral thalamus, temporal cortex and supramarginal gyrus (see Supplementary Figure S1 and Supplementary Table S1). For the opposite contrast (young > older), there was higher BOLD activity in the right frontal inferior operculum, left temporal pole and middle temporal gyrus, HC, parahippocampal cortex, fusiform gyrus, and lingual gyrus (see Supplementary Figure S2 and Supplementary Table S2). The main effect of novelty was driven by stronger activation in central superior motor areas (left and right) for new stimuli in comparison to old ones (see Supplementary Figure S3 and Supplementary Table S3). There were no significant clusters for the factor task (pFWE = 0.082, RET > STU, right supramarginal gyrus), or any of the possible interactions.


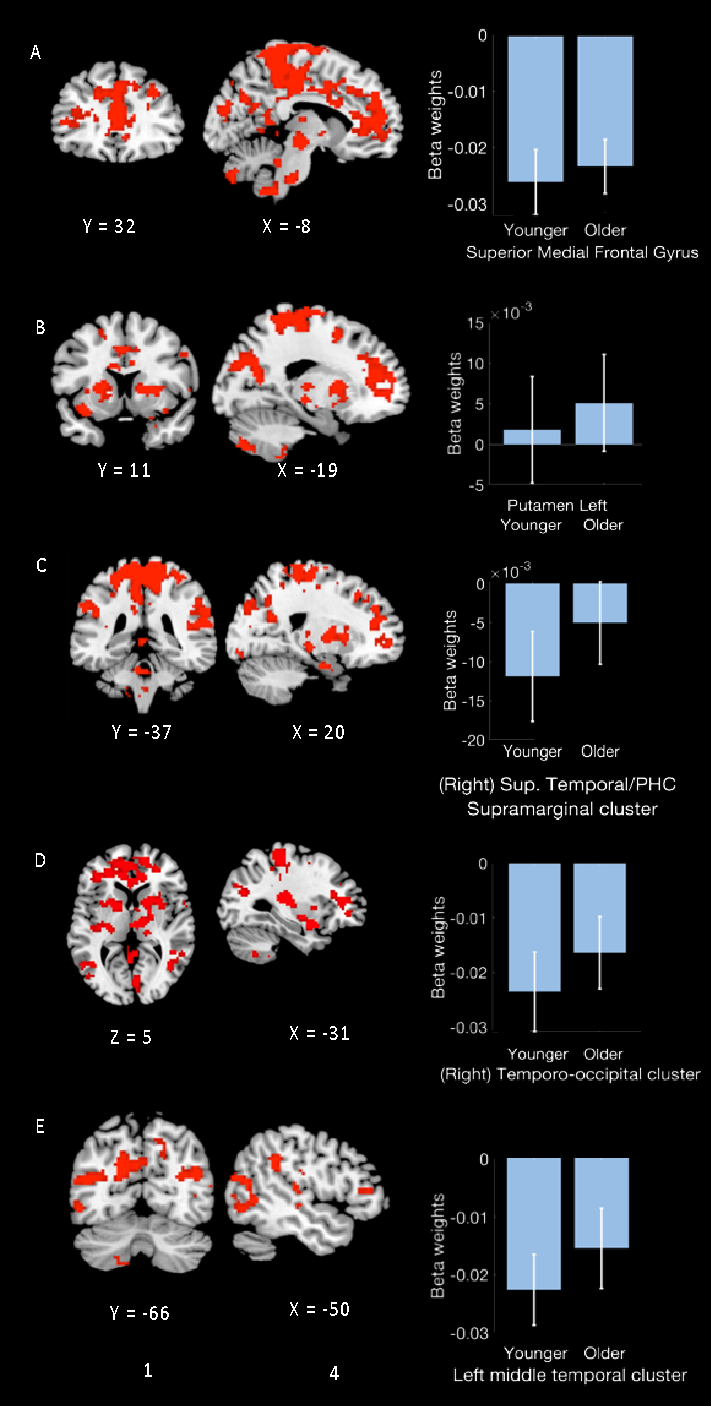


Figure S1. Age effects: higher activation in older subjects as compared to young ones (older > young). There were widely distributed activities in pre- and postcentral motor areas (bilaterally), including the superior medial frontal gyrus (A), left putamen (B), right temporo-parietal cortex, including the PHC and supramarginal gyrus (C), and temporo-occipital cortex (D), as well as the left middle temporal gyrus (E). All activation maps are thresholded at p<0.05 (FWE-corrected at cluster-level using a cluster forming threshold at voxel level of p<0.001). For a full overview of activations in this contrast, see also Supplementary Table 1. Bar plots illustrate activity in the cluster.


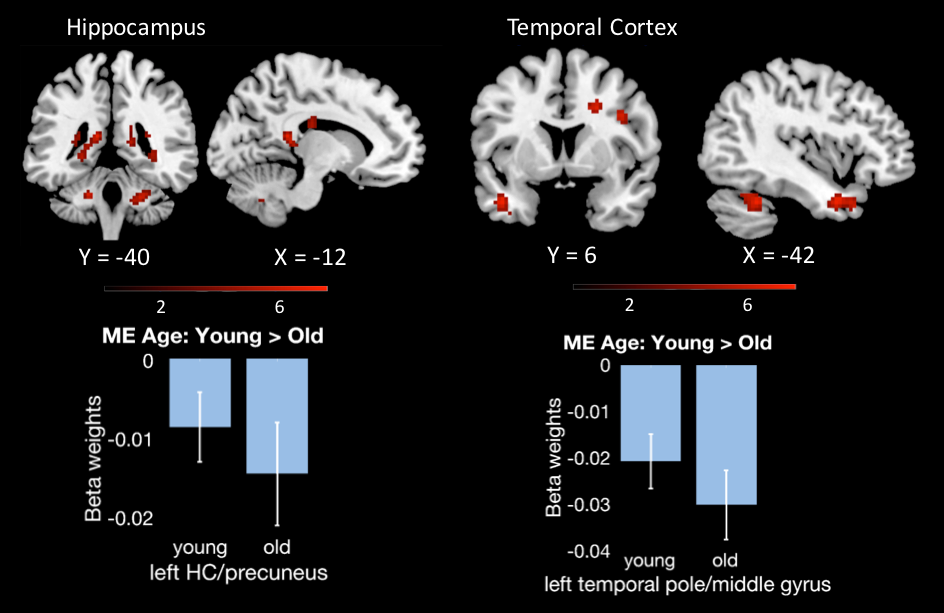


Figure S2. Age effects: higher BOLD activation in younger subjects as compared to older ones (young > older). We found significant activity within the left HC and precuneus (A), and a cluster including the left temporal pole and middle temporal gyrus (B). All activation maps are thresholded at p<0.05 (FWE-corrected at cluster-level using a cluster forming threshold at voxel level of p<0.001). For a full overview of activations in this contrast, see also Supplementary Table 2. Bar plots illustrate activity in the cluster.


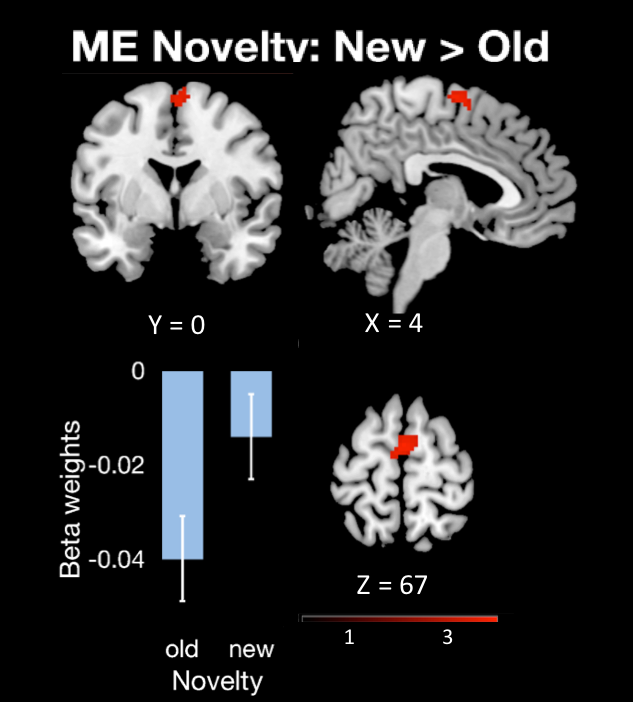


Figure S3. Novelty effects. Higher BOLD activation for new stimuli, in comparison to old ones, in the bilateral precentral gyrus, see also Supplementary Table 3. Beta weights extracted from whole cluster.


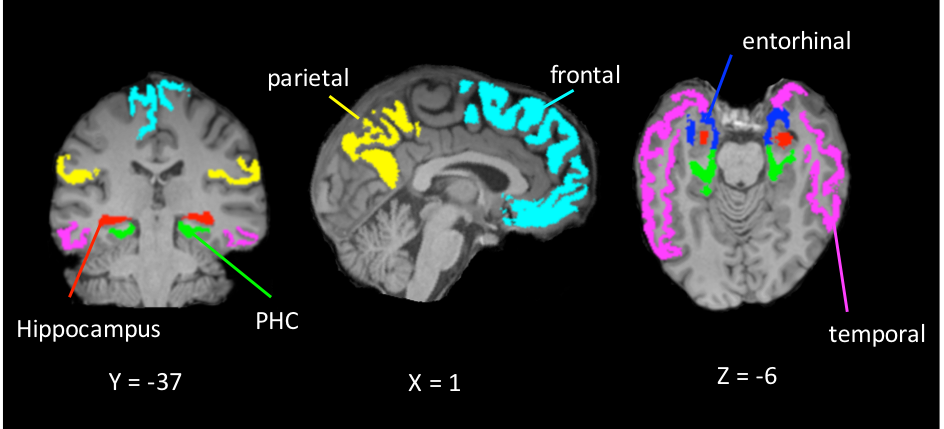


Figure S4: Location of the ROIs for the RSA.

Table S1: Trial numbers in each condition for all included participants. Trial numbers below 10 marked in red.

|  | Encoding | | | | Retrieval | | | |  |  |  |
| --- | --- | --- | --- | --- | --- | --- | --- | --- | --- | --- | --- |
|  | Old | | New | | Old | | New | |  |  |  |
| ID | Rem | Know | Rem | Know | Rem | Know | Rem | Know | CR | Unsure | Errors |
| 1 | 52 | 14 | 16 | 25 | 34 | 18 | 17 | 29 | 35 | 168 | 31 |
| 2 | 33 | 18 | 12 | 21 | 26 | 24 | 17 | 20 | 81 | 159 | 55 |
| 3 | 41 | 14 | 19 | 18 | 27 | 13 | 10 | 9 | 103 | 115 | 90 |
| 4 | 59 | 9 | 40 | 14 | 56 | 8 | 22 | 17 | 65 | 100 | 40 |
| 5 | 7 | 15 | 26 | 25 | 13 | 17 | 21 | 20 | 27 | 119 | 83 |
| 6 | 22 | 27 | 8 | 24 | 27 | 27 | 6 | 21 | 72 | 148 | 64 |
| 7 | 18 | 18 | 1 | 20 | 12 | 18 | 2 | 24 | 61 | 234 | 55 |
| 8 | 53 | 8 | 17 | 19 | 38 | 18 | 10 | 15 | 69 | 189 | 36 |
| 9 | 39 | 29 | 23 | 30 | 29 | 21 | 13 | 21 | 51 | 184 | 20 |
| 10 | 75 | 3 | 20 | 31 | 77 | 1 | 15 | 32 | 100 | 87 | 28 |
| 11 | 54 | 17 | 27 | 19 | 37 | 18 | 12 | 21 | 98 | 134 | 33 |
| 12 | 71 | 0 | 39 | 7 | 75 | 1 | 27 | 11 | 78 | 65 | 77 |
| 13 | 61 | 14 | 48 | 15 | 55 | 12 | 29 | 19 | 88 | 86 | 23 |
| 14 | 64 | 8 | 39 | 7 | 44 | 12 | 24 | 17 | 105 | 33 | 96 |
| 15 | 68 | 2 | 29 | 11 | 63 | 4 | 28 | 7 | 100 | 110 | 47 |
| 16 | 49 | 15 | 24 | 32 | 47 | 12 | 23 | 22 | 108 | 52 | 77 |
| 17 | 65 | 9 | 27 | 29 | 62 | 12 | 32 | 29 | 19 | 80 | 21 |
| 18 | 43 | 10 | 20 | 11 | 36 | 7 | 12 | 12 | 121 | 56 | 131 |
| 19 | 56 | 18 | 17 | 39 | 45 | 26 | 17 | 36 | 110 | 35 | 41 |
| 20 | 12 | 59 | 1 | 51 | 11 | 57 | 0 | 40 | 128 | 24 | 75 |
| 21 | 69 | 1 | 36 | 6 | 59 | 2 | 21 | 11 | 136 | 21 | 106 |
| 22 | 60 | 15 | 26 | 20 | 47 | 13 | 14 | 15 | 106 | 72 | 74 |
| 23 | 14 | 27 | 7 | 27 | 9 | 24 | 7 | 23 | 40 | 174 | 80 |
| 24 | 22 | 31 | 5 | 35 | 16 | 21 | 3 | 30 | 59 | 187 | 52 |
| 25 | 47 | 19 | 6 | 21 | 50 | 11 | 14 | 19 | 55 | 195 | 28 |
| 26 | 57 | 10 | 25 | 6 | 48 | 11 | 15 | 6 | 61 | 162 | 70 |
| 27 | 48 | 2 | 34 | 2 | 52 | 3 | 32 | 2 | 107 | 66 | 104 |
| 28 | 49 | 15 | 15 | 20 | 51 | 13 | 19 | 19 | 107 | 76 | 76 |
| 29 | 55 | 10 | 19 | 16 | 62 | 8 | 18 | 8 | 143 | 7 | 119 |
| 30 | 10 | 41 | 8 | 41 | 8 | 43 | 8 | 40 | 62 | 62 | 84 |
| 31 | 23 | 35 | 6 | 28 | 15 | 22 | 3 | 24 | 13 | 236 | 29 |
| 32 | 70 | 8 | 45 | 23 | 69 | 7 | 38 | 21 | 75 | 90 | 10 |
| 33 | 74 | 4 | 40 | 20 | 72 | 5 | 29 | 26 | 29 | 107 | 10 |
| 34 | 53 | 17 | 21 | 39 | 36 | 28 | 17 | 32 | 63 | 51 | 54 |
| 35 | 47 | 6 | 34 | 8 | 58 | 5 | 25 | 13 | 71 | 85 | 102 |
| 36 | 18 | 28 | 3 | 23 | 10 | 30 | 2 | 15 | 59 | 85 | 172 |
| 37 | 34 | 23 | 13 | 24 | 33 | 23 | 15 | 19 | 30 | 184 | 32 |
| 38 | 58 | 22 | 24 | 35 | 33 | 29 | 8 | 29 | 122 | 9 | 76 |
| 39 | 38 | 13 | 11 | 21 | 28 | 10 | 11 | 12 | 123 | 33 | 160 |
| 40 | 65 | 0 | 45 | 7 | 57 | 7 | 42 | 8 | 91 | 81 | 42 |
| 41 | 79 | 1 | 7 | 37 | 77 | 3 | 8 | 20 | 68 | 135 | 37 |
| 42 | 62 | 1 | 31 | 3 | 55 | 0 | 22 | 5 | 108 | 43 | 125 |
| 43 | 78 | 0 | 33 | 1 | 76 | 2 | 28 | 1 | 120 | 68 | 62 |
| 44 | 49 | 5 | 40 | 1 | 57 | 4 | 41 | 4 | 93 | 64 | 78 |
| 45 | 71 | 7 | 37 | 22 | 65 | 9 | 38 | 17 | 54 | 62 | 30 |
| 46 | 60 | 0 | 19 | 2 | 45 | 0 | 13 | 2 | 154 | 0 | 180 |
| 47 | 75 | 4 | 36 | 18 | 74 | 3 | 30 | 19 | 89 | 63 | 26 |
| 48 | 57 | 19 | 7 | 35 | 57 | 16 | 5 | 27 | 111 | 49 | 73 |
| 49 | 56 | 13 | 36 | 10 | 52 | 8 | 22 | 10 | 118 | 23 | 101 |
| 50 | 38 | 30 | 15 | 34 | 18 | 34 | 12 | 33 | 104 | 1 | 106 |
| 51 | 20 | 44 | 8 | 53 | 20 | 46 | 5 | 44 | 65 | 57 | 59 |
| 52 | 53 | 1 | 34 | 5 | 47 | 0 | 23 | 2 | 105 | 39 | 146 |
| 53 | 16 | 40 | 9 | 33 | 10 | 35 | 9 | 26 | 25 | 161 | 47 |
| 54 | 38 | 11 | 16 | 14 | 25 | 14 | 18 | 6 | 143 | 0 | 187 |
| 55 | 53 | 12 | 22 | 22 | 45 | 21 | 19 | 23 | 57 | 136 | 38 |

Table S2: Trial numbers averaged across novelty. Trial numbers below 10 marked in red. Note the large discrepancies between conditions, even if trial numbers are above 10.

|  | Encoding | | Retrieval | |
| --- | --- | --- | --- | --- |
|  | Rem | Know | Rem | Know |
| ID | averaged across novelty | | | |
| 1 | 68 | 39 | 51 | 47 |
| 2 | 45 | 39 | 43 | 44 |
| 3 | 60 | 32 | 37 | 22 |
| 4 | 99 | 23 | 78 | 25 |
| 5 | 33 | 40 | 34 | 37 |
| 6 | 30 | 51 | 33 | 48 |
| 7 | 19 | 38 | 14 | 42 |
| 8 | 70 | 27 | 48 | 33 |
| 9 | 62 | 59 | 42 | 42 |
| 10 | 95 | 34 | 92 | 33 |
| 11 | 81 | 36 | 49 | 39 |
| 12 | 110 | 7 | 102 | 12 |
| 13 | 109 | 29 | 84 | 31 |
| 14 | 103 | 15 | 68 | 29 |
| 15 | 97 | 13 | 91 | 11 |
| 16 | 73 | 47 | 70 | 34 |
| 17 | 92 | 38 | 94 | 41 |
| 18 | 63 | 21 | 48 | 19 |
| 19 | 73 | 57 | 62 | 62 |
| 20 | 13 | 110 | 11 | 97 |
| 21 | 105 | 7 | 80 | 13 |
| 22 | 86 | 35 | 61 | 28 |
| 23 | 21 | 54 | 16 | 47 |
| 24 | 27 | 66 | 19 | 51 |
| 25 | 53 | 40 | 64 | 30 |
| 26 | 82 | 16 | 63 | 17 |
| 27 | 82 | 4 | 84 | 5 |
| 28 | 64 | 35 | 70 | 32 |
| 29 | 74 | 26 | 80 | 16 |
| 30 | 18 | 82 | 16 | 83 |
| 31 | 29 | 63 | 18 | 46 |
| 32 | 115 | 31 | 107 | 28 |
| 33 | 114 | 24 | 101 | 31 |
| 34 | 74 | 56 | 53 | 60 |
| 35 | 81 | 14 | 83 | 18 |
| 36 | 21 | 51 | 12 | 45 |
| 37 | 47 | 47 | 48 | 42 |
| 38 | 82 | 57 | 41 | 58 |
| 39 | 49 | 34 | 39 | 22 |
| 40 | 110 | 7 | 99 | 15 |
| 41 | 86 | 38 | 85 | 23 |
| 42 | 93 | 4 | 77 | 5 |
| 43 | 111 | 1 | 104 | 3 |
| 44 | 89 | 6 | 98 | 8 |
| 45 | 108 | 29 | 103 | 26 |
| 46 | 79 | 2 | 58 | 2 |
| 47 | 111 | 22 | 104 | 22 |
| 48 | 64 | 54 | 62 | 43 |
| 49 | 92 | 23 | 74 | 18 |
| 50 | 53 | 64 | 30 | 67 |
| 51 | 28 | 97 | 25 | 90 |
| 52 | 87 | 6 | 70 | 2 |
| 53 | 25 | 73 | 19 | 61 |
| 54 | 54 | 25 | 43 | 20 |
| 55 | 75 | 34 | 64 | 44 |
|  |  |  |  |  |

Table S3: Clusters and peaks with stronger BOLD activation in older than in younger subjects. Peaks that did not reach significance (FWE-corrected) or lie outside the cerebrum are displayed in grey text.

|  |  | |  |  | | **statistics** | | |
| --- | --- | --- | --- | --- | --- | --- | --- | --- |
|  | **peak coordinates (MNI)** | | | **cluster** | | | **peak** | |
| peak location | x | y | z | p (FWE-corr) | # of voxels | | p (FWE-corr) | T |
| (cluster also includes vast areas of PFC and pre and post cenral gyri) | 6 | -27 | 72 | < 0.001 | 712 | | < 0.001 | 10.06 |
| superior medial frontal R | 8 | 53 | 28 |  |  | | < 0.001 | 7.71 |
| superior medial frontal L | -12 | 53 | 15 |  |  | | < 0.001 | 7.38 |
| white matter | -34 | 6 | -12 | < 0.001 | 396 | |  | 8.78 |
| Putamen L | -24 | 10 | 8 |  |  | | 0.013 | 5.26 |
|  | -24 | -17 | 8 | < 0.001 | 1676 | | < 0.001 | 8.67 |
| Thalamus R | 10 | -12 | 2 |  |  | | < 0.001 | 7.91 |
| Pallidum R | 20 | -2 | 8 |  |  | | < 0.001 | 7.07 |
| Insula R | 40 | -10 | -5 |  |  | | < 0.001 | 6.56 |
| Rolandic Operculus L | -44 | -27 | 22 |  |  | | < 0.001 | 6.22 |
| Putamen R | 20 | 13 | 5 |  |  | | 0.001 | 5.86 |
| Parahippocampal R | 20 | -7 | -22 |  |  | | 0.002 | 5.66 |
| Superior temporal R | 48 | 0 | -8 |  |  | | 0.003 | 5.63 |
| Parahippocampal R | 26 | 8 | 25 |  |  | | 0.005 | 5.49 |
| Supramarginal L | -57 | -37 | 35 |  |  | | 0.006 | 5.44 |
| Postcentral gyrus R | 66 | -14 | 20 | < 0.001 | 3060 | | < 0.001 | 7.49 |
| Superior temporal R | 58 | -40 | 20 |  |  | | < 0.001 | 7.14 |
| Superior temporal R | 46 | -30 | 18 |  |  | | < 0.001 | 6.55 |
| Middle temporal R | 46 | -54 | 12 |  |  | | < 0.001 | 6.41 |
| Superior temporal R | 53 | -34 | 18 |  |  | | < 0.001 | 6.4 |
| Superior occipital L | -17 | -70 | 30 |  |  | | < 0.001 | 6.36 |
| Insula R | 30 | -22 | 20 |  |  | | < 0.001 | 6.22 |
| Supramarginal R | 60 | -34 | 30 |  |  | | < 0.001 | 6.15 |
| Supramarginal R | 60 | -27 | 28 |  |  | | < 0.001 | 6.1 |
| Cuneus L | -7 | -87 | 20 |  |  | | < 0.001 | 6.01 |
| Cuneus L | -17 | -64 | 22 |  |  | | 0.001 | 5.99 |
| Cuneus R | 8 | -84 | 18 |  |  | | 0.001 | 5.91 |
| Angular R | 40 | -54 | 35 |  |  | | 0.001 | 5.89 |
| Cuneus R | 8 | -84 | 22 |  |  | | 0.001 | 5.89 |
| Angular R | 43 | -52 | 25 |  |  | | 0.001 | 5.74 |
| (vermis) |  |  |  |  |  | |  |  |
| Middle temporal L | -42 | -70 | 20 | < 0.001 | 456 | | < 0.001 | 6.89 |
| Middle occipital L | -37 | -77 | 28 |  |  | | < 0.001 | 6.17 |
| Middle temporal L | -54 | -64 | 18 |  |  | | 0.001 | 5.8 |
| Middle temporal L | -54 | -62 | 8 |  |  | | 0.054 | 4.88 |

Table S4: Clusters and peaks with stronger activation in younger as compared to older adults. Peaks that did not reach significance (FWE-corrected) are displayed in grey text.

|  |  |  |  | **statistics** | | | | |
| --- | --- | --- | --- | --- | --- | --- | --- | --- |
| **peak location** | **peak coordinates (MNI)** | | | **cluster** | | **peak** | |  |
|  | x | y | z | p (FWE-corr) | # of voxels | p (FWE-corr) | T |  |
| white matter/ventricle R | 38 | -2 | 28 | < 0.001 | 496 | < 0.001 | 7.75 |  |
| posterior cingulum L | -12 | -40 | 15 | < 0.001 | 213 | < 0.001 | 6.71 |  |
| HC L | -14 | -37 | 20 |  |  | < 0.001 | 6.31 |  |
| Precuneus L | -24 | -50 | 5 |  |  | 0.404 | 4.26 |  |
| middle temporal L | -42 | 6 | -30 | 0.022 | 96 | < 0.001 | 6.09 |  |
| temporal pole L | -44 | 16 | -28 |  |  | 0.007 | 5.41 |  |
| temporal pole L | -40 | 18 | -32 |  |  | 0.02 | 5.14 |  |
| inferior temporal L | -40 | -2 | -30 |  |  | 0.887 | 3.8 |  |

Table S5. Clusters and peaks with stronger activation elicited by new in comparison to old stimuli.

|  |  |  |  | **statistics** | | | |
| --- | --- | --- | --- | --- | --- | --- | --- |
| **cluster location** | **peak coordinates (MNI)** | | | **cluster** | | **peak** | |
|  | x | y | z | p (FWE-corr) | # of voxels | p (FWE-corr) | T |
| supplemental motor area L + R | 0 | -4 | 65 | 0.041 | 82 | 0.971 | 3.99 |

**Discussion**

The univariate analysis revealed differences in activation between old and new stimuli (novelty in Phase 2), as well as for younger versus older adults. Briefly, new stimuli increased activity in the bilateral precentral gyrus, an area typically related to motor functions (Stippich et al., 2002; Yousry et al., 1995) but not novelty processing. In our study, the responses to novel stimuli were slower than to familiar stimuli (data not shown), thus delaying the motor response, and therefore activity in the motor cortex. Novelty usually elicits activation in parts of the mesolimbic system, especially the SN/VTA (Bunzeck and Düzel, 2006), as well as the hippocampus (Kumaran and Maguire, 2009; Wittmann et al., 2007). However, these regions were not active in our novelty contrast. This might be related to the fact that “novel” stimuli had already been seen once in Phase 2: these stimuli were therefore not truly novel, but less familiar than “old” stimuli, that had been shown three additional times in Phase 1.

In terms of age effects, younger subjects had stronger activation in the left HC, Precuneus, middle temporal gyrus, and temporal pole (see Figure S2). All of these areas have been associated with memory processes (see main text), which is in line with the behavioral finding that younger adults generally showed higher memory accuracy than older adults. In the reverse contrast, older adults exhibited stronger activation in vast parts of the motor cortex, left Putamen and mainly right-hemispheric temporal areas. In aging brains, stronger activation of brain areas has been linked to compensatory processes (Cabeza et al., 2018, p. 201), and general reductions of specificity, especially in prefrontal regions (Morcom and Henson, 2018). Specifically, to retrieve items in Phase 3, older adults appear to recruit further brain areas, especially on the right hemisphere, while still not reaching the younger adults´ performance level and exhibiting de-differentiation patterns in terms of hemispheric asymmetries (Cabeza, 2002; Reuter-Lorenz and Park, 2010).

**References**

Bunzeck, N., Düzel, E., 2006. Absolute coding of stimulus novelty in the human substantia nigra/VTA. Neuron 51, 369–379.

Cabeza, R., 2002. Hemispheric asymmetry reduction in older adults: the HAROLD model. Psychology and aging 17, 85.

Cabeza, R., Albert, M., Belleville, S., Craik, F.I., Duarte, A., Grady, C.L., Lindenberger, U., Nyberg, L., Park, D.C., Reuter-Lorenz, P.A., 2018. Maintenance, reserve and compensation: the cognitive neuroscience of healthy ageing. Nature Reviews Neuroscience 1.

Kumaran, D., Maguire, E.A., 2009. Novelty signals: a window into hippocampal information processing. Trends in cognitive sciences 13, 47–54.

Morcom, A.M., Henson, R.N., 2018. Increased prefrontal activity with aging reflects nonspecific neural responses rather than compensation. Journal of Neuroscience 38, 7303–7313.

Reuter-Lorenz, P.A., Park, D.C., 2010. Human neuroscience and the aging mind: a new look at old problems. The Journals of Gerontology: Series B 65, 405–415.

Stippich, C., Ochmann, H., Sartor, K., 2002. Somatotopic mapping of the human primary sensorimotor cortex during motor imagery and motor execution by functional magnetic resonance imaging. Neuroscience letters 331, 50–54.

Wittmann, B.C., Bunzeck, N., Dolan, R.J., Düzel, E., 2007. Anticipation of novelty recruits reward system and hippocampus while promoting recollection. Neuroimage 38, 194–202.

Yousry, T.A., Schmid, U.D., Jassoy, A.G., Schmidt, D., Eisner, W.E., Reulen, H.-J., Reiser, M.F., Lissner, J., 1995. Topography of the cortical motor hand area: prospective study with functional MR imaging and direct motor mapping at surgery. Radiology 195, 23–29.
